# Impact of fed-batch process intensification on the productivity and product quality of two CHO cell lines expressing unique novel molecular format proteins

**DOI:** 10.1007/s00449-024-02997-3

**Published:** 2024-04-23

**Authors:** Nicolas Q. Wolnick, Marissa R. Dickson, Thaddaeus A. Webster, Roger P. Connolly, Nancy Fernandes, Vesela Encheva, Hunter Crittenden, Jessica Hodgkins, Brian C. Hadley, Gabriella Palermo, Shannon J. Hendrick, Roy A. Newell, Genevieve Gray, Christian Siltanen, Julia Armstrong, Brandon J. Downey, Carrie Mason

**Affiliations:** 1 Research and Development, Lonza Biologics, Bend, OR USA; 2 Research and Development, Lonza Biologics, Portsmouth, NH USA; 3 Research and Development, Lonza Biologics, Slough, UK

**Keywords:** Novel molecular format (NMF), Bispecific antibody (BsAb), Fc-fusion antibody, High inoculation density (HID), Process intensification, Biomanufacturing

## Abstract

While monospecific antibodies have long been the foundational offering of protein therapeutics, recent advancements in antibody engineering have allowed for the development of far more complex antibody structures. Novel molecular format (NMF) proteins, such as bispecific antibodies (BsAbs), are structures capable of multispecific binding, allowing for expanded therapeutic functionality. As demand for NMF proteins continues to rise, biomanufacturers face the challenge of increasing bioreactor process productivity while simultaneously maintaining consistent product quality. This challenge is exacerbated when producing structurally complex proteins with asymmetric modalities, as seen in NMFs. In this study, the impact of a high inoculation density (HID) fed-batch process on the productivity and product quality attributes of two CHO cell lines expressing unique NMFs, a monospecific antibody with an Fc-fusion protein and a bispecific antibody, compared to low inoculation density (LID) platform fed-batch processes was evaluated. It was observed that an intensified platform fed-batch process increased product concentrations by 33 and 109% for the two uniquely structured complex proteins in a shorter culture duration while maintaining similar product quality attributes to traditional fed-batch processes.

## Introduction

In the past decades, the demand for protein therapeutics has grown exponentially; biopharmaceutical product approvals between the United States and Europe jumped from 9 prior to 1989 to 184 between the years of 1990 to 2009, and to more than 303 approvals in the past 13 years alone [1]. While single-target monoclonal antibodies (mAbs) have long been the cornerstone of protein therapeutics, recent advancements in immunotherapy have propelled the exploration of novel molecular format (NMF) proteins. NMF proteins can be generally classified as complex protein structures, often composed of IgG fragments or other protein moieties, capable of binding to more than one target. Examples include Fc-fusion antibodies, single chain fragment variable proteins, and bispecific antibodies. NMFs, and in particular bispecific antibodies (BsAbs), show potential for the treatment of a variety of diseases due to their ability to interact with multiple unique epitopes simultaneously [2]. Due to their unique binding abilities, BsAbs offer multiple advanced interaction mechanisms which previously could not be achieved with homodimer mAbs [3]. Currently, while only nine bispecific proteins are FDA approved, over 100 BsAbs are in various stages of clinical development, underlining a surging interest in NMF proteins across the industry [4].

As the demand for therapeutic proteins continues to rise, biomanufacturers face an evolving set of challenges. First, achieving economic feasibility of large-scale production requires highly efficient processes which bolster protein yields and lower the cost of goods (CoGs). In recent years, intensified fed-batch processes have gained interest for the sizable boost in protein yield when compared to conventional fed-batch processes [5–13]. Typically, this is achieved through a high inoculation density (HID) approach, often involving a perfused N–1 predecessor. Perfusion processes leverage continuous feeding and waste removal via a cell retention device to rapidly attain high cell densities. The high cell density perfused N–1 culture then acts as an inoculum source and enables inoculation densities far higher than previously attainable for fed-batch cultures at the manufacturing scale. Intensified fed-batch processes have reported substantial increases in product concentration and space–time yield (STY) compared to traditional low inoculation density (LID) fed-batch processes. For example, several authors report a greater than 70% improvement to STY and/or product concentration using an intensified fed-batch approach [5–13]. These significant product yield improvements when using the intensified fed-batch process model have been widely documented for traditional mAb production and have thus become an increasingly popular strategy among biomanufacturers.

However, while intensified fed-batch processes have been transformative for homodimer mAb production, similar benefits for NMFs expressed through a single, recombinantly engineered cell line in intensified fed-batch processes have been less thoroughly explored. Several authors have reported increased productivity of BsAbs using perfused production bioreactor processes compared against LID fed-batch cultures [14–16]. While perfused production processes do show promise for increased productivity, as well as reducing CoGs, this cell culture approach has not overtaken fed-batch as the most commonly utilized process type for biomanufacturing. Furthermore, to the authors’ knowledge, the benefits of an intensified fed-batch process with regards to NMF production have not been widely studied.

One additional hurdle to overcome during NMF production in fed-batch culture is the well-known risk of incorrect chain pairing during subunit assembly [2, 3, 17–23]. From a biomanufacturer’s point of view, incorrectly assembled chain pairings represent missed opportunities to generate the correct product, and are detrimental to the net yield and potentially patient safety. Fortunately, innovative technologies such as CrossMAb™ (Roche) and ‘Knob-into-Hole’ have vastly improved the rate of correct chain pairings, though undesirable byproduct isoforms or incomplete pairings could theoretically still occur for some NMF structures [2, 3, 17–23]. Moreover, NMFs have been reported to frequently have high levels of undesirable product quality (PQ) attributes such as aggregation, fragmentation, and clipping in a molecule-specific manner [14, 20, 24–30]. The added structural intricacies of many NMFs, in contrast to mAbs, complicate the biomanufacturing of a consistent product and have potential to further decrement protein yields. In addition, fed-batch HID cultures could contribute their own challenges that may exacerbate the product quality hurdles observed with NMF production. With increases in protein concentration comes increased interactions between protein molecules, reducing the energy barrier for aggregation [29, 31]. Likewise, a greater peak concentration of cells in an HID process leads to a potential increase in the concentration of proteases in culture at time of harvest, which in turn may lead to protein fragmentation [32]. Considering the expanding interest in complex biotherapeutic molecules alongside the growing adoption of intensified fed-batch processes for large scale manufacturing, the impact of process intensification on the product quality of NMFs in fed-batch HID cultures remains an important and unanswered question. In this study, the productivity and product quality of two NMF proteins, a CrossMAb^™^ technology-formatted BsAb and a monoclonal antibody with an Fc-fusion protein, in both intensified and traditional fed-batch processes were compared. We demonstrate increased protein yields in the HID cultures for both NMF products, along with many comparable product quality metrics between intensified and non-intensified fed-batch processes.

## Methods

**Fig. 1:**
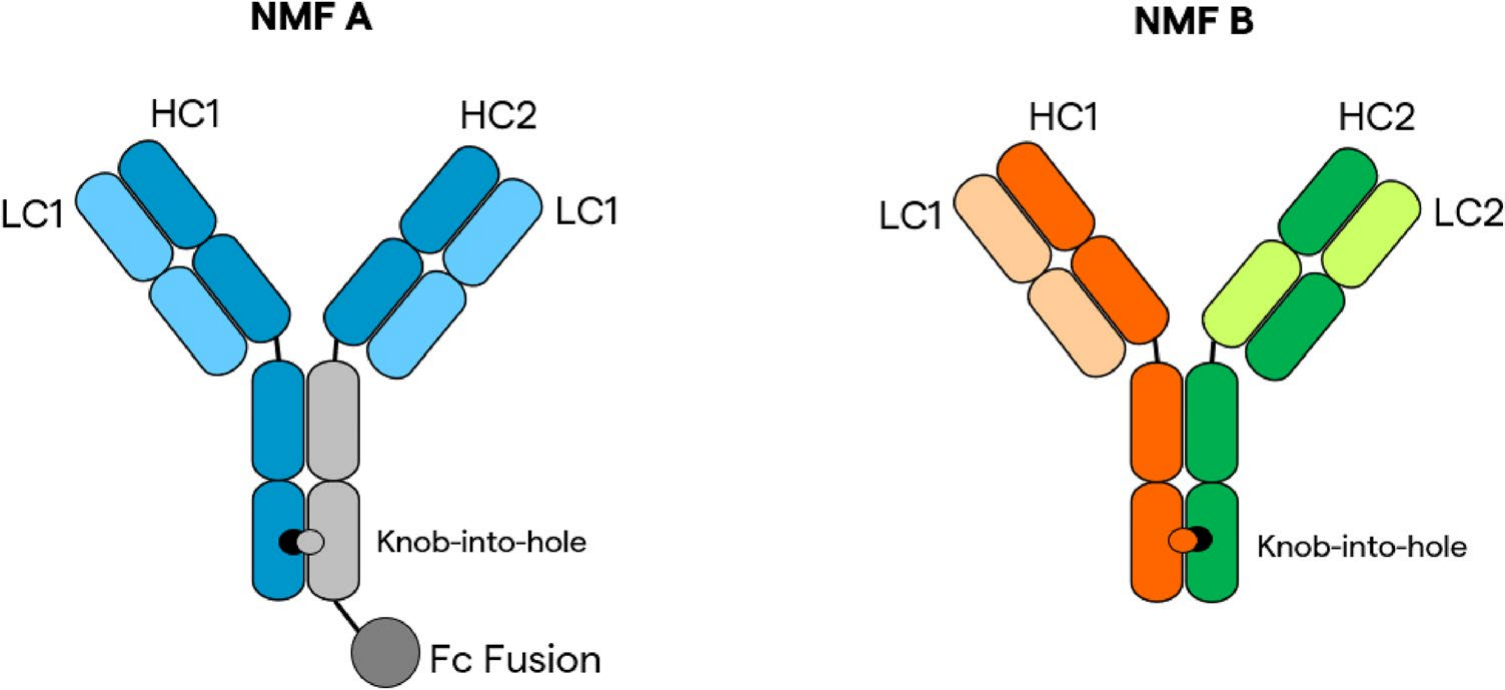
Diagram of products NMF A and NMF B

CHO clones (Lonza) expressing NMF A and NMF B (the structures of which can be seen graphically represented in Fig. 1) were cultured on two separate Lonza platform processes: a fed-batch non-intensified platform and a fed-batch intensified platform with a high inoculation density, preceded and enabled by a perfused N-1 bioreactor culture.

### N-1 and production reactors and conditions

Inoculum cultures prior to the N-1 stage for all culture iterations were expanded using Lonza’s proprietary, chemically defined inoculum media before reaching sufficient cell density and volume for N-1 inoculation.

LID platform process N-1 bioreactors were inoculated at a target viable cell concentration of 0.33 × 10^6^ cells/mL and grown for 4–5 days to a target concentration of 4.5 × 10^6^ cells/mL before being used to inoculate the production LID bioreactors. LID N-1 cultures for NMF A were grown in shake flasks, while LID cultures for NMF B were grown in 5 L Applikon STRs. The HID platform process cultures used perfused N–1 predecessor cultures to feed and concentrate cells to target transfer concentration of 50.0 × 10^6^ cells/mL in < 8 days. A Repligen alternating tangential flow (ATF) filter was used to retain cells in the bioreactor while continuously removing waste through a permeate line. Perfusion media was fed starting on day 3 of culture on the basis of inline capacitance readings at 1000 kHz, using a feed rate equation with a set capacitance-specific perfusion rate (CapSPR), feeding a specified unit of feed per unit of capacitance. A setpoint bioreactor weight was maintained through continuous removal of cell-free waste via PID control of the permeate line pump. A Kaiser Raman RXN2 system with a 785 laser, coupled with 220 or 420 mm Endress + Hauser probes were installed inline in the perfused N-1 bioreactors to measure glucose concentrations, which were maintained at a designated setpoint using the control strategies outlined in Table 1. Applikon glass STRs with a working volume of 2 L were used for all perfused N-1 cultures.

**Table 1:** Process conditions summary

Product	Working volume [L]	Production N bioreactor platform process	Predecessor (N–1) bioreactor mode	Feed control method	Supplemental Feed A normalized acceptable control limits	Supplemental Feed B normalized acceptable control limits	Supplemental Feed C Normalized Acceptable Control Limits
NMF A	1	LID	Shake flask	On–off	Continuous adjustments to maintain normalized phenylalanine to within 0.75 < x < 2.5	Continuous adjustments to maintain normalized methionine to within 0.75 < x < 2.5	Continuous adjustments to maintain normalized glucose to within 0.67 < x < 2.33
NMF A	1	LID	Shake flask	On–off			
NMF A	1	HID	Perfused N–1 (STR)	On–off			
NMF A	1	HID	Perfused N–1 (STR)	On–off			
NMF B	5	LID	STR	PID			
NMF B	5	LID	STR	PID			
NMF B	5	HID	Perfused N–1 (STR)	PID			
NMF B	5	HID	Perfused N–1 (STR)	PID			

LID production bioreactors were inoculated at a target viable cell concentration of 0.5 × 10^6^ cells/mL. pH was controlled at a setpoint of 6.90 at time of inoculation. The pH setpoint was raised to 7.10 partway through the culture and was maintained there until harvest on day 15. Temperature was initially controlled at a setpoint of 36.5 °C, and was lowered to a setpoint of 33 °C partway through the culture and maintained there until harvest. The downward temperature shift in the LID process is intended to boost culture productivity and maintain high cell viabilities until harvest. Dissolved oxygen was controlled to a setpoint of 60% of air saturation for the duration of culture.

HID production reactors were inoculated at a target viable cell concentration of approximately 10.0 × 10^6^ cells/mL. The pH was initially controlled at a setpoint of 6.90 at inoculation, then raised to a setpoint of 7.10 partway through the culture, where it was maintained until harvest on day 12. Temperature was initially controlled at a setpoint of 36.5 °C, then lowered to a setpoint of 33 °C partway through the culture and maintained there until harvest. The downward temperature shift in the HID process is intended to boost culture productivity and maintain high cell viabilities until harvest. Dissolved oxygen was maintained at a setpoint of 40% of air saturation for the duration of the culture.

The Kaiser Raman system and Endress + Hauser probes as previously described were used to measure metabolites inline via multivariate models estimating glucose, phenylalanine, and methionine. Further details regarding the development of the models governing this feeding strategy can be found in two publications by Webster et al. describing model development for both LID and HID cultures [12, 33]. Control of Supplemental Feeds A, B, and C (controlled to setpoints for phenylalanine, methionine, and glucose, respectively) was initiated shortly after inoculation. Supplemental Feeds A and B were fed as needed to maintain setpoints of phenylalanine and methionine, respectively. A supplemental glucose solution was fed as required to maintain a glucose concentration setpoint in the cultures. Due to control strategies available at different sites, NMF A cultures used on–off control for feeding, while NMF B cultures operated under PID control for feeds. Normalized concentration range limits for the supplemental feeds are listed in Table 1.

LID cultures were sampled for product quality and harvested before reaching 70% viability and before exceeding 15 days of culture. HID cultures were sampled for product quality and harvested before reaching 70% viability and before exceeding 12 days of culture.

All STR bioreactors used a sodium carbonate and sodium bicarbonate alkali solution to maintain pH control as necessary throughout culture. Gibco^™^ FoamAway^™^ irradiated animal origin-free antifoam was added as necessary via bolus addition to reduce foaming in bioreactors.

### Daily sample analysis

Offline samples were removed aseptically daily from production cultures and measured for offline pH, metabolites, and cell concentration. Daily metabolite measurements were analyzed via Nova Biomedical BioProfile^®^ Flex2 Analyzer, while cell concentration was measured via the Nova Biomedical BioProfile^®^ Flex2 CDV module for NMF A, and Beckman Coulter’s Vi-CELL^™^ XR machine for NMF B. Cell culture supernatant (CCS) was analyzed for product concentration, as well as residual amino acid analysis. Measurements for residual phenylalanine and methionine concentrations in daily culture samples were measured via high performance liquid chromatography (HPLC).

### Product quality samples and analytical methods

Culture samples for product quality analysis were taken on production harvest days, occurring on day 12 for HID cultures and day 15 for LID cultures. Samples were centrifuged, and CCS was purified over a 0.2 µm filter. Product quality CCS samples were then further purified over a column of MabSelect^™^ SuRe^™^ Protein A resin in a 5 mL HiTrap^™^ column from Cytiva^™^ Life Sciences on an ÄKTA purification system, using Lonza proprietary buffers, and aliquoted for further analytic testing.

Ultra high-performance liquid-chromatography mass spectrometry (LC–MS) for analysis of potential chain mispairing of NMF molecules was performed using a Waters quadrupole time-of-flight (QToF) Xevo^®^ G2 mass spectrometer system. Intact mass analysis was conducted on product quality samples from the day of harvest. Samples were diluted to 1 mg/mL with LC/MS-grade water, then digested with 2 µL of enzyme peptide: *N*-glycosidase F (PNGaseF) per 100 µg protein overnight at 37 °C to deglycosylate the antibody.

**Fig. 2:**
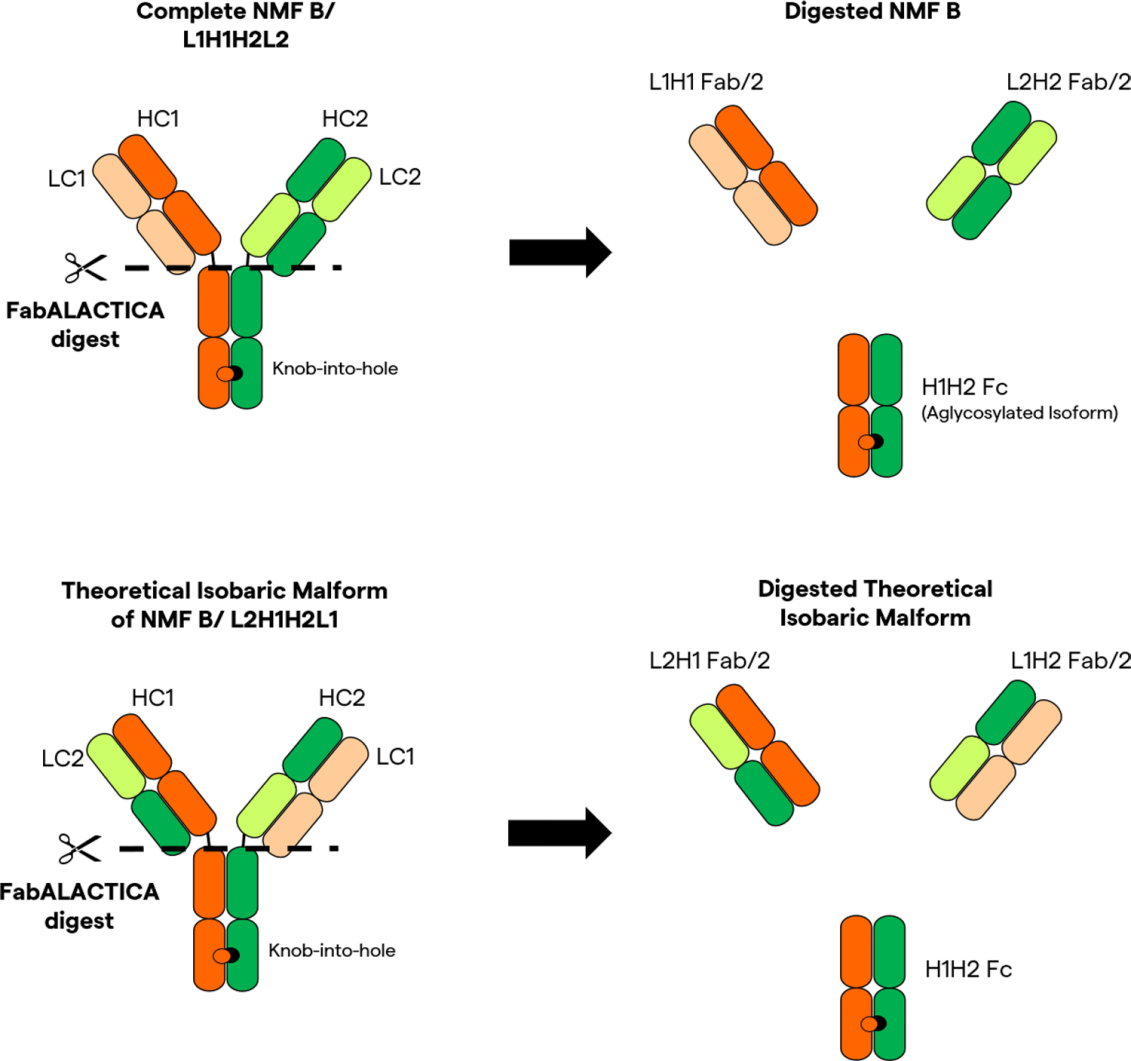
Diagram of NMF B Processing with FabALACTICA

Further mass spectrometry subunit analysis was performed on NMF B samples to determine if chain mispairing occurred. In a secondary step to further differentiate the theoretical isobaric variant from the correctly paired NMF B molecule, a cysteine protease (FabALACTICA) digest was used to cleave the antigen-binding fragments (Fab) portions from the Fc domain of the molecule above the hinge region (Fig. 2). The digested NMF B samples were then bound to a desalting column to wash off salts in an organic mobile phase, then eluted off the column. The desalted molecules were then ionized and run through the Xevo^®^ G2 system. Parent masses were deconvoluted using Byos (Protein Metric Inc.) software.

Reduced and non-reduced product analysis for protein purity was performed using capillary electrophoresis sodium dodecyl sulfate (CE-SDS). Non-reduced analysis used an SDS buffer with the addition of iodoacetamide. Reduced analysis used an SDS buffer with the addition of 2-mercaptoethanol in order to reduce the disulfide bonds of the analyzed proteins. A PA800 Plus capillary electrophoresis system from SCIEX was used to separate and detect reduced and non-reduced product sample components.

Size variant analysis to detect the proportion of monomer, aggregates, and fragments in product quality samples was conducted via size exclusion chromatography (SEC) using an Agilent 1260 Infinity HPLC system. A Tosoh TSK G3000SWXL separation column was used to separate species of different molecular weights, and protein elution from the column was detected by UV absorption at 280 nm. UV absorption peaks areas were integrated to determine the relative amounts of monomer, aggregates, and fragments detected off of the SEC column.

Oligosaccharide profiling was completed using the GlyX method, a plate-based assay for *N*-linked glycan mapping. *N*-glycan profiling was completed using the Gly-X *N*-Glycan Prep with InstantPC Kit from Agilent. Deglycosylation occurs via the enzyme Rapid PNGaseF, and released glycans are fluorescently labeled with 2-aminobenzamide. *N*-glycan profiles were then obtained by running the fluorescently tagged samples on a Waters ultra-performance liquid chromatography (UPLC) system. The relative percentage of each eluted glycan was calculated based on the area of the glycan peak relative to the total integrated peak area.

## Results

### Culture performance in LID and HID bioreactors

**Fig. 3:**
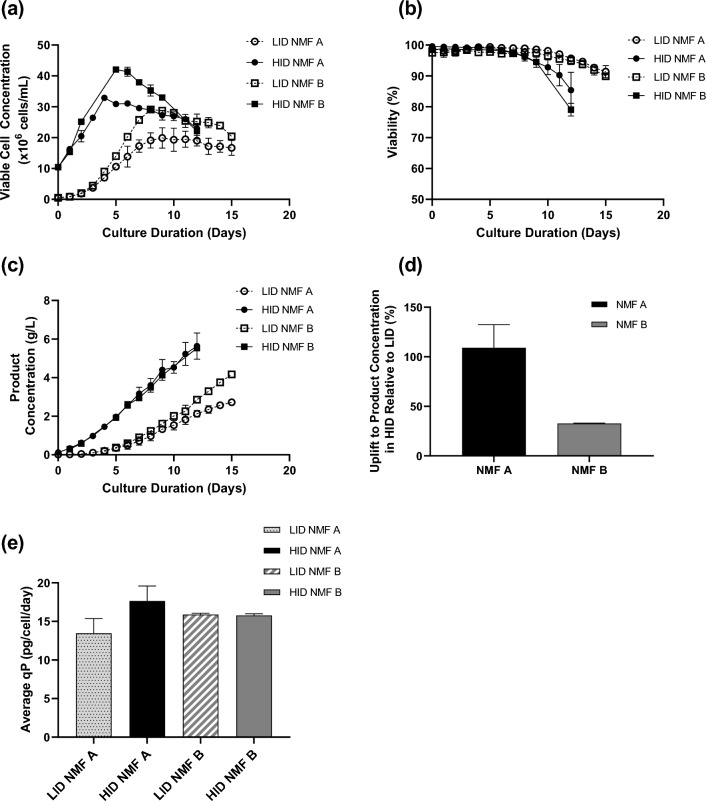
**a **Viable cell concentration, **b** Viability, **c** and Product concentration in averaged LID and HID processes for both NMF A (hollow and solid circles) and NMF B (hollow and solid squares). **d** Percent average uplift to product concentration between the HID cultures and LID cultures for both NMF A (black solid bar) and NMF B (gray solid bar). **e** Average cell-specific productivities for LID NMF A (gray dotted bar), HID NMF A (black solid bar), LID NMF B (gray striped bar), and HID NMF B (gray solid bar). Error bars represent one standard deviation from the mean (*n* = 2)

LID NMF A cultures achieved peak viable cell concentrations between days 10 and 12 of culture, with concentrations of 22.0 and 17.8 × 10^6^ cells/mL (Fig. 3a). Viable cell concentrations for LID NMF A cultures on day 15 at time of harvest reached 18.4 and 15.0 × 10^6^ cells/mL. NMF B LID batch cultures reached peak viable cell concentrations of 26.5 and 29.7 × 10^6^ cells/mL on day 6 of culture, and subsequently decreased in concentration until reaching concentrations of 19.8 and 21.0 × 10^6^ cells/mL, respectively, at the time of reactor harvest on day 15 of culture. Culture viability percentages for both sets of LID cultures remained high and above acceptable viability limits throughout the 15 days of the process, with viabilities of NMF A in LID cultures at harvest above 92.2%, while viabilities of NMF B in LID cultures at harvest were both above 89.7% viability (Fig. 3b).

In HID cultures, cell concentration peaked at higher concentrations and earlier, with NMF A HID cultures reaching their maximum concentrations on day 4, and NMF B HID cultures reaching maximum concentrations on day 4–5. Similarly to their LID counterparts, NMF A HID cultures had lower peak measured viable cell concentrations than NMF B cultures, with NMF B cultures reaching concentrations of 42.7–43.2 × 10^6^ cells/mL at their maximum, while NMF A cultures reached maximum VCCs of 32.2–33.6 × 10^6^ cells/mL. HID cultures for both NMF A and B both saw decreased viability on the day of harvest in comparison to LID cultures; the lowest NMF A HID culture decreased to a cell viability of 81.3% on day of harvest, while the lowest viability reached by NMF B in HID cultures was 77.6%. All HID cultures remained above acceptable viability limits for the HID platform process. Commonly referenced waste metabolite (lactate, ammonium) concentrations were generally similar between the HID and LID processes for both NMF A and NMF B (data not shown). Harvest day culture lactate levels were maintained below 3 g/L for all but one culture (HID NMF B day 12, data not shown). The 3 + g/L lactate concentration measured on day 12 of one HID NMF B culture likely had minimal impact on culture performances as harvest cell viability & product concentration were similar between biological replicates (Fig. 3b, c). Harvest day culture ammonium levels were similar between the HID and LID processes for both NMF A and NMF B, falling between 4.5 and 6 mM (data not shown).

The forward-shifted and elevated peak VCC of the HID cultures reflected the HID process goal of mitigating the days necessary for exponential phase growth in LID cultures and jump-starting the production phase of cell culture. This was confirmed in the product concentration profiles, where an increased and early production of NMF A and B in HID cultures was observed compared to LID cultures. While LID cultures reached averaged concentrations of 2.6 and 3.7 g/L for NMFs A and B, respectively after 15 days of culture, HID cultures achieved an average 5.3 and 4.9 g/L, respectively in 12 days (Fig. 3c). For NMF A, this equated to a 109% (± 23.4%) average uplift in product concentration; NMF A saw a lower average uplift in product concentration from LID to HID cultures of approximately 33% (± 0.45%) (Fig. 3d). Moreover, HID cultures expressing NMF A had an approximately 30% higher average cell-specific productivity (qP) than the LID cultures (Fig. 3e). There was a negligible (< 1%) difference in the average qP between the LID and HID cultures expressing NMF B.

### Product quality comparison for NMF A

Product quality attributes of protein harvested from HID and LID cultures were measured to evaluate the impact of process intensification on chain-pairing/product assembly, aggregation, purity, charge variant profile, and glycosylation. The LID process specifies product harvest on day 15, while this harvest occurs on day 12 in the HID process. While the three-day difference in culture duration has the potential to impact product quality, a harvest-day comparison of the attributes is most representative of how the processes would be performed and analyzed at the manufacturing scale.

**Fig. 4:**
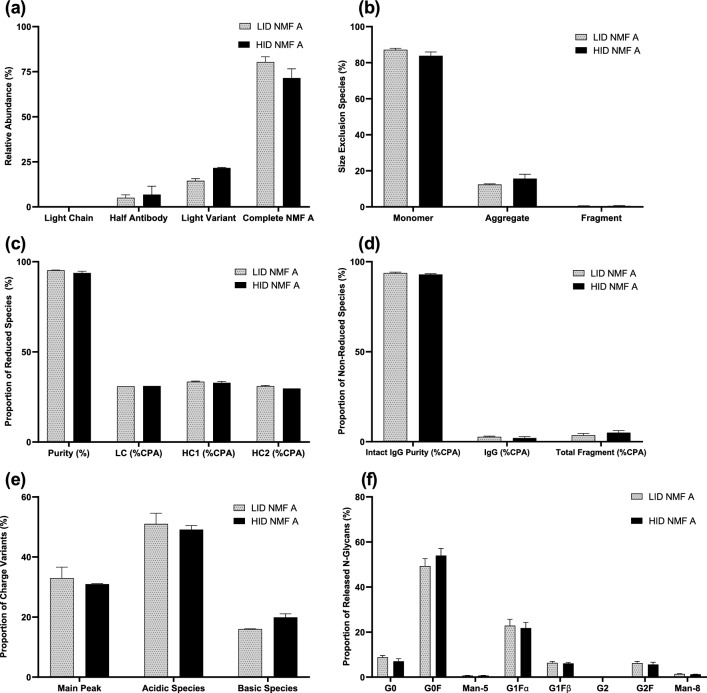
Product quality attributes for cultures expressing NMF A in the 12-day HID process (black solid bars) and 15-day LID process (gray dotted bars). **a** Relative abundance of NMF A subunits, **b** Size exclusion, **c** Reduced species, **d** Non-reduced species, **e** Charge variants, **f** *N*-Glycans. Error bars represent one standard deviation from the mean (*n* = 2)

Intact mass spectrometry (MS) analysis confirmed that for both LID and HID cultures, the majority of the harvested product was correctly paired NMF A molecules at 80.4 and 71.5% of relative abundance per measured intensity, respectively (Fig. 4a). Table 2 below displays graphic representations of the NMF A structure, as well as potential incorrectly paired subunits and variants that can be detected using intact MS. In the intensified process cultures, the intensity proportion of the light variant of the molecule, which lacks the Fc-fusion protein on the heavy chain, increased from an average of 14.5–21.6%. Light chain fragments were detected in minimal proportions in both LID and HID cultures. Size exclusion chromatography showed consistently high percentage of monomer in LID and HID cultures (Fig. 4b). Some aggregation was detected, with a small increase in average percentage from 12.0 to 15.4 with process intensification. Negligible fragment levels were detected, with neither process average exceeding 0.3%. Reduced and non-reduced purity analysis showed largely similar purity levels, subunit proportions, and intact IgG purities between the LID and HID processes with no differences larger than 1.5% (Fig. 4c, d). Charge variant analysis demonstrated minimal changes, with a decrease of approximately 2% to the proportion of main peak and acidic species, alongside a 4% increase to the observed basic species (Fig. 4e). The rank-order of the charge variants was unchanged between the LID and HID processes expressing NMF A, with acidic species being of the highest proportion followed by main peak and basic species. Analysis of the released N-glycans between the processes showed similar proportions of measured glycoforms (Fig. 4f). The proportion of G0F species increased and the proportion of G0 species minimally decreased in the HID cultures compared to the LID cultures.

**Table 2:** NMF A subunits diagram

Light chain	Light variant half antibody	Light variant	Theoretical heavy variant	Complete NMF A
				

### Product quality comparison for NMF B

Product quality attributes of protein harvested from HID and LID cultures were measured to evaluate the impact, if any, of process intensification on chain-pairing/product assembly, aggregation, purity, charge variant profile, and glycosylation. Harvest samples were collected and analyzed for PQ from the final process day in both HID and LID cultures with the intention of best representing the comparison that would be made at the manufacturing scale.

**Fig. 5:**
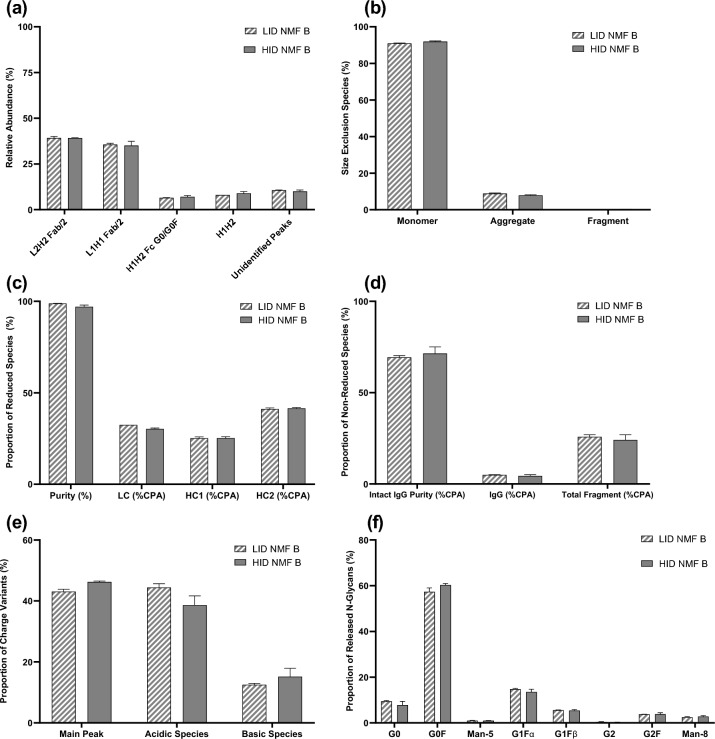
Product quality attributes for cultures expressing NMF B in the 12-day HID process (gray solid bars) and 15-day LID process (gray striped bars). **a** Relative abundance of FabALACTICA Digested NMF B subunits, **b** Size exclusion, **c** Reduced species, **d** Non-reduced species, **e** Charge variants, **f** *N*-Glycans. Error bars represent one standard deviation from the mean (*n* = 2)

For the LID and HID cultures expressing NMF B, analysis of chain-pairing/product assembly was performed via mass spectrometer in combination with FabALACTICA digest. Similar relative abundance levels of the post-FabALACTICA digest subunits displayed in Table 3 were observed in LID and HID cultures expressing NMF B. Additionally, similar levels of unidentified peaks were observed between both processes (Fig. 5a). Size exclusion analysis showed comparable abundance of monomer and aggregate species with approximately a 1% increase to monomer species and 1% decrease to aggregate species observed in the HID cultures compared to the LID cultures (Fig. 5b). Reduced purity analyses of NMF B found high levels of purity as well as largely similar proportions of light chain (LC), heavy chain 1 (HC1), and heavy chain 2 (HC2) between the LID and HID cultures (Fig. 5c). Though NMF B has two unique light chains, the method used for reduced purity analysis in this study was not sensitive enough to enable differentiation of the light chains in this assay. Non-reduced purity analysis showed low, though comparable, levels of intact IgG at approximately 69 and 71% for the LID and HID processes, respectively (Fig. 5d). Interestingly, charge variant analysis found a change in the rank-order of species variant abundance between the processes (Fig. 5e). Greater proportions of main peak and basic species were observed in the HID cultures alongside a decrease in acidic species levels. This yielded a change in the rank-order, with main peak overtaking acidic species as the most abundance species in the HID cultures compared to the LID cultures. Analogously to the changes observed during *N*-glycan analysis of NMF A, glycoform comparison across processes for NMF B was largely similar (Fig. 5f), with HID cultures expressing NMF B having slightly increased levels of G0F species and slightly decreased levels of G0 species when compared to the LID cultures.

**Table 3:** FabALACTICA digested NMF B subunits diagram

L1H1 Fab/2	L2H2 Fab/2	H1H2 Fc G0/G0F	H1H2

## Discussion

Recent advancements in immunotherapy have rejuvenated interest in bispecific antibodies, and novel molecular format proteins as a whole, due to their expanded therapeutic potential when compared to traditional mAbs. To meet the growing market demand for protein therapeutics, many biomanufacturers have developed high inoculation density fed-batch processes that substantially improve space time yield and maintain similar product quality attributes when compared to low inoculation density fed-batch processes. However, intensified fed-batch processes have yet to be thoroughly evaluated for the production of more complex proteins. This study evaluated and compared the productivity and product quality attributes of two NMF proteins cultured in LID and HID platform processes.

HID cultures are designed to improve overall volumetric productivity, primarily by increasing viable cell concentrations throughout the lifespan of the N production bioreactor. In this evaluation, the HID cultures expressing NMF A consistently maintained higher cell densities than the LID cultures (Fig. 3a). HID cultures expressing NMF B also maintained higher cell densities than the LID cultures for all but the final 2 days of the process. On average, the HID cultures achieved uplifts to product concentration of 109% (± 23.4%) and 32.6% (± 0.45%) for NMF A and NMF B, respectively when compared to the LID processes (Fig. 3d). The improvements to product yield for both NMF A and NMF B align with previously reported uplift ranges observed during the production of mAbs in intensified processes. The difference in uplift between the two NMF products tested may be due to changes in the cell-specific productivity rates between the HID and LID cultures. Both positive and negative changes to qP during process intensification have been previously observed during the production of traditional mAbs. The HID process described in this study aims to increase productivity primarily through sustained higher VCCs, without intentionally affecting cell-specific productivity. Unexpectedly, a 30% increase was observed between the average cell-specific productivity rates between the LID and HID cultures expressing NMF A (Fig. 3e). In contrast, there was a negligible (approximately 1%) decrease to the average cell-specific productivity rates between the LID and HID cultures expressing NMF B (Fig. 3e). While it is possible that qP changes directly in response to culture seeding density, the degree of qP change appears to be clone dependent, as observed by the difference in HID cell-specific productivities between NMF A and NMF B. This observation may suggest that some clones are more ‘fit’ for some processes than others, though this has yet to be thoroughly investigated. The exact mechanisms that explain variance in cell-specific productivity in the context of process intensification remain unknown, but would likely be fruitful to explore in future studies.

Comparable product quality attributes between processes, especially during the production of complex proteins, is of critical importance. The generation of protein product that does not meet established standards for product quality both negatively impacts the net yield of the process and poses a potential safety risk for the patient. As previously mentioned, NMFs can inherently bring additional PQ challenges due their asymmetrical design. Chain mispairing and increased propensities for aggregation, fragmentation, and clipping represent a handful of the additional obstacles worth consideration during the production of complex proteins. While many of the potential PQ obstacles are common between these NMFs, the unique structural differences between the protein products evaluated in this study require that their product quality attributes be assessed individually. For example, NMF A is a monospecific antibody with dual modes of action due to a protein fused to the Fc region of one heavy chain (Fig. 1). The two arms that make up the Fab region of NMF A are identical, eliminating the risk of chain mispairing. However, the presence of a Fc-fusion protein opens up the possibility of forming off-target heavy and light variant isoforms. Theoretical heavy variant isoforms contain Fc-fusion proteins bound to both heavy chains on a single IgG molecule, while light variant isoforms have no Fc-fusion proteins attached (Table 2). NMF B falls into the category of a traditional bispecific antibody, with variant light and heavy chain pairs capable of binding unique epitopes. Though NMF B was designed using technologies such as ‘Knob-into-hole’ and CrossMAb^™^ which greatly mitigate the risk of chain mispairing, due diligence as a biomanufacturer requires product chain pairing be analyzed. The following paragraphs examine the product quality attribute changes observed during process intensification for each NMF separately.

Many of the assessed product quality attributes for NMF A cultures were similar between the LID and HID processes. Reduced purity, non-reduced purity, charge variant, and *N*-glycan analyses had minimal differences by process, suggesting that process intensification had little to no impact on these attributes for NMF A (Fig. 4c–f). However, assembly analysis via mass spectrometer showed a decline of approximately 9% in the relative abundance of completely formed NMF A alongside an increase of approximately 7% to the relative abundance of light variant between the LID and HID cultures (Fig. 4a). The presence of light variants of NMF A could be due to the phenomenon known as clipping, in which protein moieties fused to the antibody are enzymatically cleaved from the structure. Clipping of Fc-fusion proteins is somewhat commonly observed, and has been hypothesized to be due to protease activity and/or defective protein folding [26–32, 34]. The increased abundance of light variant NMF A species in the HID cultures may be partially explained by the declining viabilities observed in the final few days of the HID process. It is possible that as the viable cell densities and viabilities decline from their peaks, intracellular protease enzymes are released from dying cells, which then clip the product. Additionally, size-exclusion analysis of the HID NMF A cultures demonstrated a small decrease to the monomer species abundance and a small increase to the aggregate species abundance. Aggregation is also a well-documented occurrence during the production of complex proteins, and it is possible that the higher protein concentrations generated in the intensified process increased the likelihood of product aggregation [15, 24–27]. In the context of industrial biomanufacturing, though Fc-fusion protein clipping and increased aggregation are certainly undesirable, the levels of aggregation and clipping observed for NMF A in the intensified fed-batch process were likely small enough to be offset by the large increase to productivity.

For the LID and HID cultures expressing NMF B, analysis of chain-pairing/product assembly via intact mass spectrometer confirmed that the majority of the protein isoforms expressed were either the complete, correctly paired BsAb, or an unlikely theoretical isobaric formation in which the light chains had swapped, which could not be discriminated by the intact method (data not shown). The FabALACTICA digestion was then performed to determine the chain pairing of the identified majority protein isoforms. FabALACTICA digestion aims to break the antibody into three subspecies, as outlined in Fig. 2, which allows for more specific identification and quantification of the chain pairs present in the original whole-antibody structure. Analysis of the post-FabALACTICA digest samples showed no peaks that correspond to the theoretical Fab region subunits with L1H2 or L2H1, suggesting that the monomer detected is most likely the correctly paired variant (Figs. 2, 5a). Unexpectedly, full-length H1H2 species lacking light chain pairs (Table 3) were detected at relative abundance levels of approximately 8–9% in both LID and HID processes (Fig. 5a). The existence of such subspecies post-FabALACTICA digest suggests that the intended separation of the antibody structure into three parts was not fully achieved prior to analysis. Additionally, the presence of the multiple unidentified peaks, which were collectively summed in Fig. 5a, also supports the idea that complete digestion was not achieved. However, the summed unidentified peaks are present at much lower percent abundances than the correctly paired and fully digested NMF B subunits for both processes. Therefore, with the consistent percent abundance of correctly paired subunits between both the LID and HID cultures, it can be concluded that the intensification of the process did not negatively affect the pairing of heavy and light chains of NMF B. Lack of negative impact attributable to process intensification is further supported by the results of the non-reduced purity analysis, in which the proportion of intact IgG were within 1.5% between the LID and HID processes (Fig. 5d). While very similar between processes, NMF B showed high levels of fragmentation, negatively impacting the abundance of intact species and theoretical yield of product as a whole. The exact cause for high fragmentation levels of bispecific antibodies is unknown, and likely varies by the structure and stability of the molecule. One previous study hypothesized that chemical reduction of bispecific molecules with engineered disulfide bonds lead to higher levels of fragmentation than has historically been observed during the production of monospecific antibodies [28]. Another study found that a CHO protease caused fragmentation and product degradation of three different BsAbs [32]. In this study, similarly high levels of fragmentation in both the LID and HID processes indicate that the root causal issue is not related to process intensification, but potentially an inherent trait of NMF B when cultured in a fed-batch process. The shifting in rank-order during charge variant analysis may suggest that there are post-translational modification differences occurring between the LID and HID processes. Sialyation, deamidation, and several other post-translational modifications can cause the formation of acidic variants. The LID process had approximately 6% higher proportions of acidic species compared to the HID process for NMF B (Fig. 5e). Differences in post-translational modifications can have varying level of impact on the efficacy of the product, and may depend on the location of the modification. In some cases, no effect is observed. In others, the binding affinity or potency of the molecule is affected [35].

This study aimed to investigate the impact of fed-batch process intensification on the productivity and product quality of CHO cell lines expressing two unique NMFs. Cultures expressing each NMF were run in duplicate in both the LID and HID platform processes. The data generated in this study demonstrated that production of NMFs via fed-batch process intensification is a viable strategy for improving productivity. Though some differences in measured product quality attributes were observed for both NMF A and NMF B, the differences were often overshadowed by commonly observed PQ obstacles that have been previously reported on during the production of complex proteins. Other researchers have reported a decrease to clipping and aggregation for complex proteins through the use of perfusion technology in the production bioreactor. Several previous publications have hypothesized that the continuous clearing of waste products from the culture, decreased bioreactor protein concentration, and/or improvements to endoplasmic reticulum stress levels were responsible for their observed improvements to these PQ attributes [15, 16, 26]. In contrast, fed-batch processes accumulate waste byproducts and product resides in increasing concentrations in the bioreactor environment from the time of cell secretion to harvest. Future experiments investigating PQ differences across processes during the production of complex antibodies may find that a time course of samples throughout the duration of each process could highlight the dynamics, if any, of product quality attributes such as clipping and aggregation. Our results suggest that the fed-batch process intensification generally has low impact on many of the product quality attributes of typical concern during biomanufacturing. Those that were clearly negative, such as the decrease to the relative abundance of completely assembled NMF A (Fig. 4a) are likely to have their detriments mitigated by the large increase to volumetric productivity that comes with fed-batch process intensification.

## Data Availability

Research data are not shared.

## Abbreviations

***ATF***: Alternating tangential flow
***BsAb***: Bispecific antibody
***CapSPR***: Capacitance-specific perfusion rate
***CCS***: Cell culture supernatant
***CHO***: Chinese hamster ovary
***CoGs***: Cost of goods
***HID***: High inoculation density
***LID***: Low inoculation density
***mAb***: Monoclonal antibody
***NMF***: Novel molecular format
***PAT***: Process analytical technology
***STY***: Space–time yield
***VCC***: Viable cell concentration
***qP***: Cell-specific productivity
